# The effect of football (soccer) heading on gross and fine motor control in women

**DOI:** 10.3389/fspor.2025.1620442

**Published:** 2025-07-02

**Authors:** Jac L. Palmer, Bert Bond, Alex Woodgates, Jacob Jack, Oliver Smail, Ryan Baker, Genevieve Williams

**Affiliations:** ^1^ Exeter Head Impact, Brain Injury and Trauma (EXHIBIT) Research Group, Public Health and Sport Sciences, University of Exeter, Exeter, United Kingdom; ^2^ The Vascular Research Group, Public Health and Sport Sciences, University of Exeter, Exeter, United Kingdom

**Keywords:** soccer, mild traumatic brain injury (mTBI), motor control, cognitive function, visual-motor processing

## Abstract

**Introduction:**

Heading is an integral component of football, but concerns remain about its impact on brain health. This study examines the acute effects of heading on gross and fine motor control as a measure of the motor-cognitive function of women footballers.

**Methods:**

The heading protocol for this study represented the typical exposure to headers experienced in the women's game: one every 10 min, for one hour, replicating a corner kick. A sample of 19 female collegiate football (soccer) players participated in two sessions: a control session, and a heading intervention. Gross motor control was assessed via measures of sway during standing balance, and fine motor control was evaluated using a precision finger grip task.

**Results:**

Results showed no significant changes in gross motor control, based on postural sway parameters. However, significant alterations were observed in fine motor control in the tremor frequency (0–4 Hz band) of precision gripping, indicating a potential change in motor-cognitive function following the heading task.

**Discussion:**

The findings suggest that exposure to the number and type of headers that might be performed over a typical football match does not impair standing balance, but it may affect fine motor control. Future research should look to incorporate brain imaging and electrophysiological measures to further understand the mechanisms underpinning changes in fine motor control performance after heading.

## 1 Introduction

Heading is a crucial skill in football (soccer), contributing to 32.5% of all contestable goals scored at the 2022 Women's World Cup (1). A meta-analysis by McCunn et al. (2) concluded that the average impact forces encountered during heading are below established thresholds for brain injury. While the acute brain function response to head impacts is of particular interest, there is also concern about the potential chronic effects of exposure to repeated head impacts [e.g., (3)]. Nowinski et al. (3) reviewed existing evidence and suggested that repeated head injuries may be linked to an increased risk of neurodegenerative diseases, though more research is needed to establish causality definitively. Acutely, DiVirgilio et al. (4) showed immediate and measurable negative alterations in brain function characterised by reduction in memory function and increased corticomotor inhibition, following a “standard” bout of football heading. Increasing our understanding of the acute effects of sport-related head impacts on brain function is critical for immediate and long-term athlete health monitoring in sport.

Motor control assessments have been used to provide evidence of neurological changes that occur following head impacts. Measures of gross motor control, in the form of standing balance, have been utilised on the principle that standing balance relies upon an ensemble of complex mechanisms, including cortical and subcortical pathways (5, 6). Bonke et al. (7) reviewed studies on football heading and found mixed results: a number of studies reported impaired standing balance following heading [e.g., (4, 8–10), while others found no significant changes [e.g., (11–14)]. The Bonke et al. (7) review concluded that future research should prioritise female athlete populations in future football heading research. MRI studies have demonstrated that female athletes are more susceptible to negative white matter microstructure alterations following football heading, compared to male athletes (15). Additionally, research by Bretzin et al. (16) and Caccese et al. (17), found that female athletes experienced greater head accelerations during heading compared to male athletes. These findings highlight female athletes as a target group for further research, to establish the effect of football heading on brain function.

Fine motor skills have also been extensively utilised in the assessment of brain function, and may provide functional insights related to the alterations in brain microstructure observed following heading. Previous studies have applied precision finger gripping tasks in distinguishing people with attention deficit hyperactivity disorder (18–20), autism (21, 22), and Parkinson's disease (23). Employing neuroimaging, precision gripping tasks have been shown to evoke activity in superficial brain areas relevant to head injuries such as the ventral pre-motor area, the rostral cingulate motor area, and at several locations in the posterior parietal and prefrontal areas across both hemispheres (24–26). More recently, Studenka and Raikes (27) showed that males and females with a previous history of concussion performed significantly worse in precision grip tasks (specifically, the accuracy of the force applied) compared to individuals with no concussion history. In addition, females who had suffered two or more concussions showed reduced signal complexity and lower spectral power in precision grip force outputs between 8 and 12 Hz compared to controls, indicative of impairment in visual processing and predictive motor control systems. Parr et al. (28) utilised a hand grip force matching task following 12 headers in a five-minute period. This study found significantly improved force-matching performance following a heading intervention. Therefore, precision finger-gripping tasks could be utilised to highlight subtle changes in cognitive-motor function following heading in football.

Despite extensive research, there is no consensus on the effects of football heading on standing balance. The inconsistencies in findings may be driven by the varied interventions and data collection methods that have previously been employed. This study aims to assess the gross and fine motor control changes following a heading intervention that is reflective of match conditions in women's football. The purpose of this study was to provide evidence as to whether match condition heading scenarios acutely influence standing balance and fine motor function.

## 2 Materials and methods

### 2.1 Participants

Ethical approval was obtained from the University of Exeter Sport and Health Sciences Ethics Committee (2012-A-01), and participants provided voluntary written informed consent prior to any data collection. Nineteen female football players (defenders = 10, midfielders = 5, forwards = 4), were recruited from the University of Exeter Football team. The participants (age = 21. 9 ± 3.1 years, playing experience = 11.7 ± 5.4 years, stature = 1.64 ± 0.05 m, mass = 64.3 ± 7.9 kg) were screened for any contraindicators prior to performing the heading protocol. The screening required players to be uninjured, free from concussion for a minimum of two months, and have no history of migraines or seizures.

### 2.2 Protocol

All participants attended a familiarisation session in which they experienced all testing protocols. After the familiarisation, participants were invited to the laboratory for two data collection sessions separated by a minimum of seven days. The data collection sessions consisted of a heading protocol or a control condition which were performed in a counterbalance order. During the heading protocol, match-play heading frequency was replicated by the heading of the football, six times every hour with a single ball delivered at 10-min intervals (29–31). Participants remained seated after each header. A motorised ball launcher (Ball Launcher Pro Trainer, Globaltec Innovation LTD, Chester, England), positioned 15 m from the participants with an official size 5 UEFA Women's Champions League football and always pressurised to the regulation 12 psi, was used to standardise the ball speed and trajectory. A ball velocity of 40 ± 5 km/h was selected to reflect match play heading intensity, in line with previous literature (4, 10, 17). To ensure ecological validity, participants were instructed to deliberately head the ball toward a researcher positioned perpendicular to its trajectory. This approach was designed to simulate a cross into the box, where an attacker might head the ball toward the goal, or a defender might clear it to safety. During the control condition participants were seated and rested for the same time period of 1 h. Measures were taken before and following the heading protocol or the control condition in the same order: firstly, cerebrovascular assessments reported in Jack et al. (32), followed by the balance and grip force tasks reported here. Participants finished their headers (outdoors), and then walked back into the lab. For both the heading and control condition, balance and finger grip tasks were performed after cerebrovascular assessments (32) which were commenced at the 1-h time point and lasted approximately 30 min.

### 2.3 Grip force task

Our precision finger grip test (PFG) was administered, with the participant seated with a sternum to screen distance of ∼50 cm, facing a 14″ (35 cm × 17.5 cm) LCD (HP, Palo Alto, California) monitor displaying the force target. Participants held a bespoke 3D printed precision finger grip device, with a centrally mounted low profile load cell between the thumb and index finger of their right hand. Participants completed the PFG trials in the vision conditions that they would generally assume for playing football. Force target and PFG force data were shown and collected in a bespoke application in the LabView software by National Instruments (National Instruments, Austin, Texas). Participants performed trials with continuous immediate feedback throughout, referred to as full vision (FV) trials. Trials with partial feedback requiring them to perform precision gripping from memory referred to as non-vision (NV) trials. Participants completed one FV followed by two NV trials, with target force set to a consistent value of 13 N, equating to the ∼30% of the sex-aggregated mean maximal voluntary contraction (MVC) force output as presented by Dottor et al. (33). This threshold was selected to elicit substantial effort while remaining within the participants' functional capacity. For the FV trials, the moving force bar remained visible throughout the 20 s trial, providing real-time visual feedback. During the NV trials, the force bar was visible for 8 s and subsequently disappeared for the last 12 s of the trial, and participants were instructed to continue producing force at the target level until the trial ended.

A colour change signified the start of the trial, with a visual cue on screen of a box turning from red to green. The colour change was at a consistent time interval which allowed for reaction time (RT) could be calculated as the difference between the visual cue and force onset. PFG force was recorded via a 250 N load cell (TE Connectivity, Schaffhausen, Switzerland) amplified via a National Instruments DAQ board (National Instruments, Austin, Texas). PFG force was recorded at 1,600 Hz via Lab View and visualised as a bright blue block, which was removed for the NV trials.

### 2.4 Balance task

For the balance task, ground reaction forces were collected whilst participants stood in a double-leg stance with their hands on their hips, under two conditions: eyes open and eyes closed. In the eyes open condition, participants focused on an “X’ marked 4 meters ahead at eye level. Throughout the balance tests participants aimed to remain as still as possible while keeping their feet together and hands on top of the iliac crest. The double-leg stance has been shown to be sensitive to changes in postural control acutely, following head impact events (34, 35). Data were collected for 30 s per trial. Ground reaction forces were collected using an AMTI force plate (Watertown, Massachusetts, MA, USA) with a sampling rate of 1,000 Hz.

### 2.5 Data processing

Raw PFG transducer data was processed using custom code written in Python 3.9, utilising the following libraries: pandas for data manipulation (36), NumPy for numerical operations (37), Matplotlib for plotting and visualisation (38), and SciPy for signal processing and scientific computation (39). Data were filtered with a low-pass 4th order Butterworth filter with the cut-off set at 20 Hz and down-sampled to 100 Hz. RT was manually digitised from waveform data. The first second following the stimulus and the last second of the PFG trial were removed to allow for a settled force trace. Data was split into the first seven seconds with visual feedback and the next 12 s without visual feedback (NV). All metrics were calculated separately for both FV and NV data. Values of route mean square error (RMSE), sample entropy, and tremor frequency were calculated from the settled force trace. PFG RMSE was calculated as the root mean squared error of the applied force from the target line and values were presented as the percentage difference from the target. PFG multiscale sample entropy (MSE) was calculated by using code based on the method by Costa et al. (40). Sample entropy was calculated across seven scales and summed to give a value of MSE. Oscillations in force production (OFP) tremor frequency was calculated by conducting a power spectral density estimate via Welch's method for each second of settled data; power in the frequency bands 0–4 hz and 4–8 hz were summed and then averaged across all one-second bins.

The FV and NV phases of the cropped, filtered, and down-sampled PFG waveforms were isolated. Six one-second bins were created for the FV and NV phases. Mean and standard deviations for each of the one-second bins were calculated and plotted. The mean and standard deviation for RMSE for the entire force interval in the full-vision condition were also calculated.

Balance test centre of Pressure (CoP) data were also analysed using custom code written in Python. The raw data were filtered using a low-pass 4th-order Butterworth filter with a cut-off frequency of 20 Hz. The range of motion (ROM) in the mediolateral (ML) and anterior-posterior (AP) directions was calculated as the difference between the maximum and minimum CoP positions. The average CoP velocity was computed based on the mean point-to-point displacement divided by time. The MSE for CoP was calculated as described above for postural sway fluctuations during both the eyes-open and eyes-closed conditions.

### 2.6 Statistical analysis

Descriptive statistics were calculated with pre- and post-means, and standard deviations were presented for the control and intervention conditions. Change values were calculated for pre- and post-measures. Difference testing was performed for the change values between conditions. Statistical analyses were performed using SSPS (version 29, IBM) with statistical significance predefined at a threshold of *p* < 0.05. The assumptions of sphericity and normality were assessed using Mauchly's and the Shapiro–Wilk tests, respectively. *T*-tests and Wilcoxon signed-rank tests were used for normally distributed and non-parametric data, respectively. Cohen's *d* was calculated to assess the magnitude of the difference between conditions. Values were interpreted in accordance with conventional thresholds: 0.2 indicating a small effect, 0.5 a medium effect and 0.8 or greater a large effect (41).

## 3 Results

The results for the PFG are shown in Table 1. The heading intervention elicited a decrease in OFP in the 0–4 Hz band for the NV PFG, conversely, an increase in OFP in the 0–4 Hz band was observed following the control. A statistically significant difference [*p* = 0.03, *t* (19) = 2.37] was observed for OFP for the 0–4 Hz band for the pre- to post-change between control and intervention for NV PFG.

**Table 1:** Mean ± SD for pre-, post- and change data for precision finger gripping task results for heading intervention and control condition. Statistical difference testing was performed between control and intervention change values.

Variable	Intervention			Control			Statistics	
	Pre	Post	Change	Pre	Post	Change	*p*	*d*
Reaction time (s)	0.16 ± 0.06	0.18 ± 0.11	0.01 ± 0.10	0.17 ± 0.07	0.18 ± 0.06	0.03 ± 0.08	0.27^a^	0.12
Vision								
RMSE (%)	2.19 ± 1.80	2.25 ± 1.73	0.06 ± 1.72	2.82 ± 2.12	3.13 ± 3.40	0.46 ± 3.87	0.55^a^	−0.14
MSE	0.77 ± 0.34	0.66 ± 0.32	−0.11 ± 0.37	0.72 ± 0.41	0.61 ± 0.30	−0.06±0.35	0.65	0.11
OFP 0–4 hz (dB)	9.32 ± 1.22	9.38 ± 1.41	0.06 ± 20	9.19 ± 1.41	9.48 ± 1.24	0.72 ± 2.63	0.57	−0.2135
OFP 4–8 hz (dB)	−32.55 ± 6.60	−33.10 ± 4.31	−0.55 ± 7.42	−33.32 ± 6.07	−33.91 ± 4.38	−2.79 ± 11.67	0.93	0.15
No vision								
RMSE (%)	5.70 ± 2.48	6.68 ± 5.45	0.98 ± 5.61	6.60 ± 6.12	5.34 ± 3.77	−0.91 ± 6.83	0.86^a^	0.16
MSE	0.30 ± 0.31	0.29 ± 0.26	−0.02 ± 0.41	0.35 ± 0.29	0.24 ± 0.15	−0.08 ± 0.33	0.56	0.13
OFP 0–4 hz (dB)	7.56 ± 1.72	7.27 ± 1.82	−0.29 ± 2.67	6.54 ± 1.57	7.63 ± 1.46	1.36 ± 2.55	0.03*	−0.53
OFP 4–8 hz (dB)	−31.32 ± 8.23	−35.13 ± 4.97	−3.81 ± 8.60	−32.26 ± 5.09	−33.06 ± 5.28	−2.87 ± 10.60	0.86^a^	−0.07

^a^ Denotes non-parametric statistical analyses were performed.

* Denotes a statistically significant (*p* < 0.05) difference. Vision, visual feedback provided throughout trial duration; no vision, partial feedback provided during trial; RMSE, route mean square error of the applied force from the target line; MSE, multiscale sample entropy; OFP, oscillations in force production.

**Figure 1:**
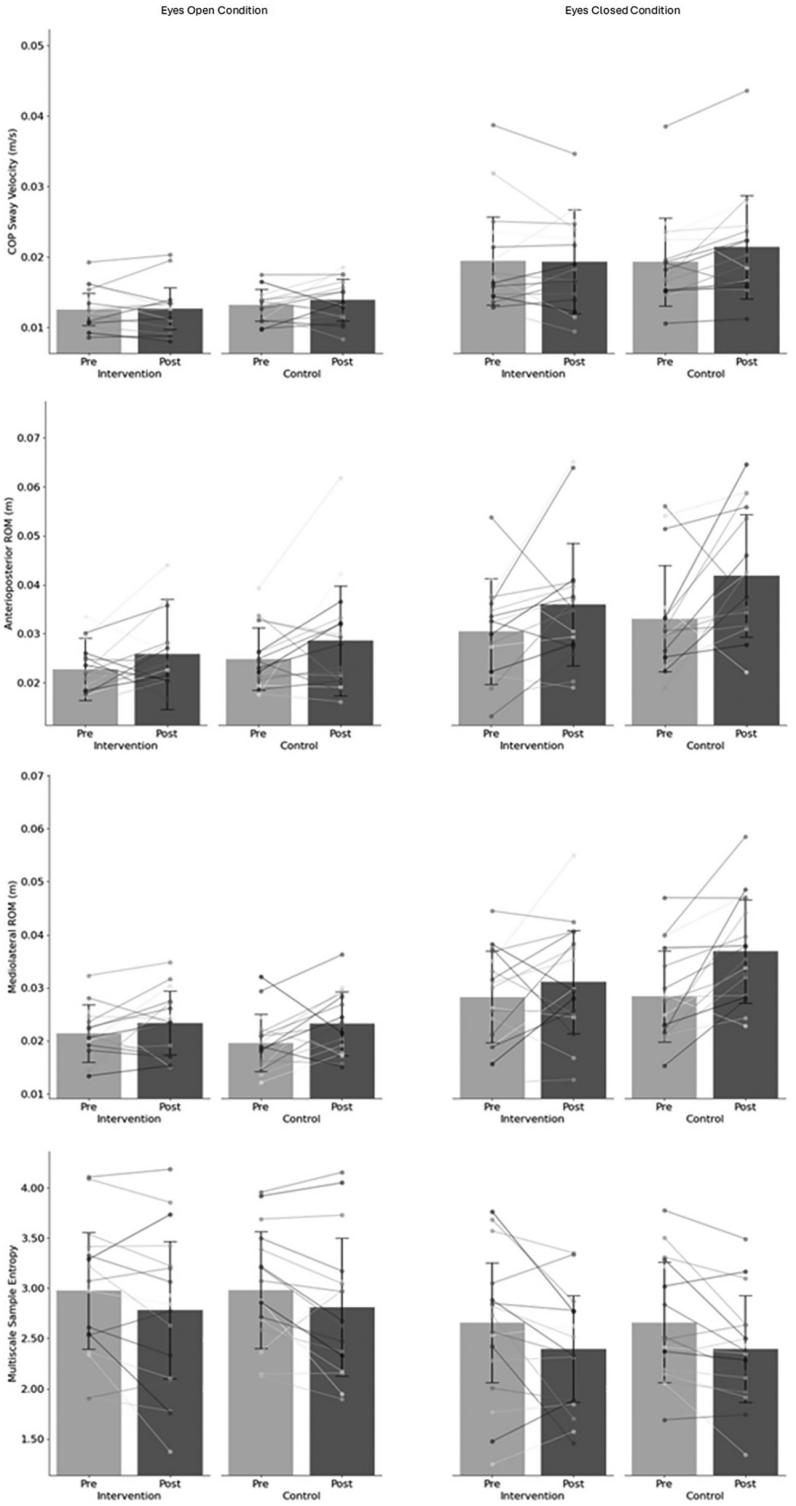
Intervention and control results for centre of pressure (COP) anteroposterior range of motion (ROM) **(a)**, mediolateral ROM **(b)**, sway velocity **(c)** and centre of pressure multiscale sample entropy **(d)** for eyes open (left) and eyes closed (right) conditions.

This study showed no significant differences between pre- and post-values of postural sway parameters in the intervention or control condition. As seen in Figure 1, COP sway velocity, as well as ML and AP ROM, were greater in the eyes-closed condition in both the intervention and control conditions.

## 4 Discussion

This study aimed to assess changes in gross and fine motor control in female footballers following a heading intervention. This study was designed to replicate the ball speeds and heading frequency observed in women's football matches. The results showed a significant difference in OFP for the 0–4 Hz band for the pre- to post-change between control and intervention. This study did, however, show that no significant changes in gross motor control, in the form of standing balance, occurred after the heading intervention.

Indices of heading dose are volume (number of headers), frequency (the rate at which headers are administered), and intensity (ball speed). In this study, female footballers performed six headers delivered at 40 ± 5 km/h, separated by 10-min intervals. At marginally increased ball delivery velocities of 48 km/h, Schmitt et al. (14) found no changes to measures of standing balance for male and female participants. They did, however, report a significant increase in concussion symptoms amongst their cohort. Although potentially ethically contentious, at greater ball delivery velocities of 80 km/h, twice that of the present study, significant reductions in verbal memory scores and reaction times were observed following the heading intervention in university age male and female football players (42). Future research should look to examine the effect of a range of realistic ball velocities ∼40 km/h as outlined in previous studies [e.g., (4, 10, 17)] during heading on acute neurological and physiological function in women's football. To further investigate the effect of match realistic heading on fine motor control, research should also look to incorporate a longitudinal approach to observing the effect of heading on motor control strategies. Collecting at intervals across hours and days following a bout of heading as well as across a season would allow us to better qualify the change in OFP observed in this study.

During the 2019 Women's World Cup, players headed the ball between 0 and 22 times per match, with an average of ∼5 ± 1 headers per match (43). McCunn et al. (2) further reported that women performed between one and nine headers per game across both matches and training sessions. In University populations mean head impacts per 90 min played were reported at 6.98 and between 6.08 and 8.31 depending on position, by McCuen et al. (30) and Lynall et al. (29) respectively. The procedures in this study aimed to mimic a 60-min training session with heading frequency aligned with previous match analyses in women's football. Previous studies utilising heading interventions with higher frequencies of ball delivery produced differing results from the present study. Relative to baseline greater sway velocity was recorded by Caccese et al. (8) in double-leg standing independent of age, sex, and concussion history when players completed 12 headers in 12 min, delivered at 40 km/h. These findings support the possible existence of critical thresholds for heading exposure.

The present study found a significant difference in tremor frequency of precision grip following the heading protocol relative to the control condition, with a medium to large effect. The change in tremor frequency may indicate an altered motor control strategy following the heading intervention. Changes in the 0–4 Hz frequency as seen in this study are associated with movements typically ∼200 ms, that require cognitive processing to control the interaction with the force target (27). Power observed in the 0–4 Hz frequency band is associated with slow time scale, sensorimotor feedback processes (44, 45). Changes in the 0–4 Hz band were not unexpected given that, refined neuromuscular innervation of the finger musculature that facilitates simple reaction time and feedback control are associated with slower fluctuations occurring <4 Hz (46). The findings of this study indicate that repeated heading of a football may induce alterations to motor control strategies during a PFG task in female football players. The current findings agree with Parr et al. (28), who collected neurophysiological and electrophysiological measures, pre- and post-20 headers over a five-minute period. Parr et al. (28) found significant changes in fine motor control during a precision force handgrip task, where they reported increased activity in the primary sensorimotor cortex responsible for right-hand contractions. The authors concluded that the recorded corticomuscular hyperconnectivity may be due to a compensatory mechanism, leading to increased brain-muscle communication. It is currently unclear if these changes may occur due to exercise irrespective of heading.

### 4.1 Limitations

The effects of heading a football on brain function remains a complex and multifaceted area of research, requiring continued investigation. Vaillancourt et al. (26) demonstrated through functional MRI (fMRI) that precision finger gripping activates the parietal and premotor cortices, regions linked to visuomotor processing. However, a notable limitation of this study was the absence of brain imaging or electrophysiological data acquisition, which could have provided a more comprehensive understanding. Incorporating such data would have enabled more precise identification of brain regions showing altered activation levels following football heading. Future studies that incorporate brain imaging, such as fMRI, or electrophysiological measures, such as EEG, will vastly enhance our understanding of the acute effects of exercise and repeated head impacts on motor control strategies.

### 4.2 Conclusion

This study suggests that a match realistic number of headers consisting of six headers in 1 h, at ball speeds representative of those recorded in the women's game does not alter measures of gross motor control. This study does suggest that heading may alter fine motor skill execution in a precision gripping task. Further studies should look to incorporate brain imaging and electrophysiological measures to further understand the mechanisms underpinning changes in fine motor control performance after heading. Since precision gripping is known to be sensitive to subtle changes in brain function, longitudinal studies to assess the effects of cumulative exposure over a season or playing career could consider employing such measures.

## Data Availability

The raw data supporting the conclusions of this article will be made available by the authors, without undue reservation.

## Nomenclature

Heading: A football technique involving the use of the head to direct the ball.
Gross Motor Control: The coordination of large muscle movements, typically involving postural control and balance.
Fine Motor Control: The coordination of small muscle movements, often evaluated through precision gripping tasks.
Precision Finger Grip Task (PFG): A task used to measure fine motor control, where participants apply specific amounts of force using their fingers.
Tremor Frequency: Oscillations in force production measured in Hz (0–4 Hz band), indicating motor function changes.
Motor-Cognitive Function: The interaction between motor skills and cognitive processes.
Postural Control: The ability to maintain balance and stability in various positions.
Ecologically Valid Protocol: A study design mimicking real-world conditions, in this case, match-play scenarios.
Force Matching Task: A task that involves matching the applied force to a target value, used in motor control assessments.
Centre of Pressure (CoP): A measure of the point where the total body pressure is applied, used in balance assessments.
Range of Motion (ROM): The distance over which movement occurs in the body, in this case, for the centre of pressure.
Reaction Time (RT): The time it takes to respond to a stimulus.
Full Vision (FV): Condition in the PFG task where continuous visual feedback is provided.
Non-Vision (NV): Condition in the PFG task where visual feedback is removed after an initial period.
Oscillations in Force Production (OFP): Repeated fluctuations in the force applied, indicating changes in motor control.
Multiscale Sample Entropy (MSE): A measure used to quantify the complexity and predictability of a time series, such as force output in a gripping task.
Sway Velocity: A measure of the speed at which the body sways during a balance task.
Anterior-Posterior (AP) ROM: Movement range in the forward and backward directions.
Mediolateral (ML) ROM: Movement range in the side-to-side directions.
Motorised Ball Launcher: A device used to standardise ball velocity and trajectory in the heading intervention.
Electrophysiological Measures: Techniques like EEG are used to assess brain function by recording electrical activity.
Corticomuscular Connectivity: Brain-muscle communication, often measured by electrophysiological tools.
Neurodegenerative Diseases: Diseases that cause progressive loss of nerve cells and brain function, potentially linked to repeated head impacts.
Neuroimaging: Techniques like fMRI used to visualise brain activity and structure.
Concussion: A mild traumatic brain injury caused by a blow to the head or body.
